# Supramolecular Gels Based on *C_3_*-Symmetric Amides: Application in Anion-Sensing and Removal of Dyes from Water

**DOI:** 10.3390/molecules29092149

**Published:** 2024-05-05

**Authors:** Geethanjali Kuppadakkath, Sreejith Sudhakaran Jayabhavan, Krishna K. Damodaran

**Affiliations:** Department of Chemistry, Science Institute, University of Iceland, Dunhagi 3, 107 Reykjavík, Iceland

**Keywords:** LMWGs, *C_3_*-symmetric amides, stimuli-responsive, water remediation, dye adsorption

## Abstract

We modified *C_3_*-symmetric benzene-1,3,5-*tris*-amide (BTA) by introducing flexible linkers in order to generate an N-centered BTA (N-BTA) molecule. The N-BTA compound formed gels in alcohols and aqueous mixtures of high-polar solvents. Rheological studies showed that the DMSO/water (1:1, *v*/*v*) gels were mechanically stronger compared to other gels, and a similar trend was observed for thermal stability. Powder X-ray analysis of the xerogel obtained from various aqueous gels revealed that the packing modes of the gelators in these systems were similar. The stimuli-responsive properties of the N-BTA towards sodium/potassium salts indicated that the gel network collapsed in the presence of more nucleophilic anions such as cyanide, fluoride, and chloride salts at the MGC, but the gel network was intact when in contact with nitrate, sulphate, acetate, bromide, and iodide salts, indicating the anion-responsive properties of N-BTA gels. Anion-induced gel formation was observed for less nucleophilic anions below the MGC of N-BTA. The ability of N-BTA gels to act as an adsorbent for hazardous anionic and cationic dyes in water was evaluated. The results indicated that the ethanolic gels of N-BTA successfully absorbed methylene blue and methyl orange dyes from water. This work demonstrates the potential of the N-BTA gelator to act as a stimuli-responsive material and a promising candidate for water purification.

## 1. Introduction

The quantity of contaminants of emerging concern (CECs) [1,2], which are chemicals and toxic materials in wastewater, has dramatically increased since the pre-industrial era. The synthetic dyes found in wastewater are mostly generated from industries such as textiles, cosmetics, paper, pharmaceuticals, and paint, and these can be considered as CECs due to their toxicity to ecological or human health [3]. The non-biodegradable nature of synthetic dyes leads to ecological problems due to their accumulation on land and in aquatic environments [4]. This problem resulted in the development of various techniques to remove dyes from wastewater [5], such as adsorption, photodegradation, oxidative or biochemical degradation, chemical precipitation, ion exchange, and electrocoagulation. Adsorption techniques can be considered among the best techniques for removing dyes from wastewater [6] because most adsorbents can be recycled due to their non-reactive nature to toxic substances. In this process, dyes interact with the adsorbent via chemical or non-bonding interactions, separating them from the mixture [7]. Several adsorbents [8] based on activated carbon, zeolites, mineral clay, chitosan, and waste biomass have been employed for the wastewater dye removal process. The drawbacks of pollutant uptake and selectivity, the cost involved in activating the adsorbent for recycling, and the enormous amount of toxic sludge generated prompted researchers to explore alternative methods for dye adsorption. Supramolecular gels based on low-molecular-weight gelators (LMWGs) [9,10,11,12,13] are excellent candidates for use in adsorption studies due to their porous networks and stimuli-responsive properties [14,15,16,17,18,19]. LMWGs display superior adsorption capacities for dyes [20,21,22,23], but the majority of LMWGs reported for the removal of dyes are metallogels or pH-dependent [14,24,25,26,27,28].

LMWGs have gained considerable attention during the last two decades as soft materials due to their intriguing potential applications, such as in stimuli-responsive materials, media for synthesis and crystallization, drug delivery, tissue engineering, and environmental clean-up, including oil spills and the removal of toxic chemicals [9,10,11,12,13]. LMWGs are fibrous networks arising from the self-assembly of gelator molecules in the presence of a solvent medium, which is stabilized by non-covalent interactions, and the solvent molecules are trapped within these networks. These networks are stabilized by various non-covalent interactions such as hydrogen bonding, van der Waals interactions, π–π stacking, and halogen bonding. The nature of these interactions is responsible for the stimuli-responsive behavior of LMWGs, where the gelation process can be turned ON/OFF in the presence of an external stimulus (temperature, pH, sound, salts/ions, or additives) [15,16,17,18,19]. LMWGs resemble protein structures in terms of solvent content and their porous nature grants better dye adsorption properties than traditional adsorbents [29]. However, predicting the formation of a supramolecular gel is a challenging task because the self-assembly process depends on the dynamic nature of the non-bonding interactions, the nature of the functional groups, and the balance between the hydrophilic and hydrophobic interactions [9,10,11,12,13]. The formation of a one-dimensional (1-D) fibrous architecture capable of entangling into a 3-D network in the presence of a solvent is considered one of the important criteria for gel network formation. Introducing hydrogen bonding groups, such as amide, urea, thiourea, amino acids, and hydrazone moieties, into LMWGs [30,31,32,33,34,35] can induce 1-D self-assembly via various non-bonding interactions, which results in a myriad of LMWGs with intriguing properties.

Several reports on LMWGs with tunable properties are based on amide moieties [36]. Amide-based compounds display complementary N−H···O=C interactions, arising from the donor (N-H) and acceptor (C=O) of the amide moieties, to form a 1-D fibril structure, and these 1-D chains self-assemble to 3-D porous architecture, within which the solvent molecules are entrapped [36,37]. These cooperative and unidirectional hydrogen bonding interactions arising from the amide units play a crucial role in the self-assembly process of LMWGs, and these moieties can interact with anions, leading to stimuli-responsive materials [15,16,17,18,19]. Supramolecular gels based on *C_3_*-symmetric amides display versatile gelation abilities due to their hydrogen-bonded helical columnar structures, which are stabilized by hydrogen bonding and π–π stacking. An example of this is benzene-1,3,5-*tris*-amide (BTA) [15,16,17,18,19]. However, *C_3_*-symmetric amides have rarely been used to remove dyes, presumably due to the absence of suitable porous architectures and a lack of selectivity for substrates [38,39]. Separation and selectivity can be improved by introducing/modifying functional groups to the supramolecular gels in order to produce LMWGs with better adsorption capacities. The post-modification of the functional groups via covalent capture can be used to tune their stability and robustness, leading to better adsorption performances [40,41]. Qiu et al. showed a folic acid-gelatin hybrid gel obtained by introducing gelatin into folic acid improved the specific surface and porosity of the hybrid gel, making it a better adsorbent for dye adsorption [41]. In this work, we have modified the *C_3_*-symmetric N-centered BTA with flexible linkers to explore its stimuli-responsive properties and ability to act as an adsorbent of both cationic and anionic dyes in water.

## 2. Results and Discussion

### 2.1. Synthesis of C_3_-Symmetric N-Centered BTA (N-BTA)

**Scheme 1:**
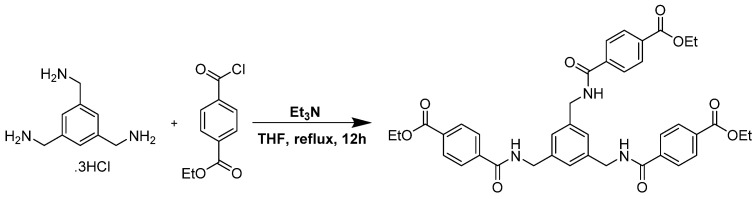
Synthesis of N-centered *C_3_*-symmetric *tris*-amide (N-BTA).

Supramolecular gels based on *C_3_*-symmetric BTA can be classified as either C=O centered or N-centered BTA molecules based on their connection to the aromatic platform, and these compounds display similar 1-D columnar structures stabilized by the hydrogen bonding interactions between the amide functionalities [42]. Haldar et al. analyzed the role of the spatial orientation of the amide bonds in the self-assembly process of C=O centered and N-centered *C_3_*-symmetric tripeptides gels and studied the dye adsorption ability of the gels [38]. The C=O-centered *C_3_*-symmetric BTA compounds (benzene-1,3,5-tricarboxylic amide) display versatile gelation abilities in a wide range of solvents, [43,44,45] but the LMWGs based on the corresponding *C_3_*-symmetric N-centered BTA are rare, presumably due to the unavailability of the amine precursor or difficulties in synthesizing benzene-1,3,5-triamine. We designed a *C_3_*-symmetric N-centered BTA (N-BTA) by introducing a methylene group between the aromatic core and the amine. We reported that adding/modifying the functional groups produces LMWGs with tuneable properties [34,46,47,48]. The *C_3_*-symmetric N-BTA was synthesized by reacting benzene-1,3,5-triyltrimethanamine with 4-(ethoxycarbonyl)benzoic acid chloride (Scheme 1). The ester group was selected because the self-assembly mode of the amide group was preserved by introducing the ester derivatives, flanking the hydrogen bonding between amide and carboxylate functionalities.

### 2.2. Gelation Studies

Solvent screening was undertaken to identify the ideal solvents/solvent mixtures for use in gelation by following a standardized protocol. The required amount of the compound was introduced into a standard 7.0 mL sealed vial, followed by the addition of 1.0 mL of the corresponding solvent. The resulting mixture was sonicated and heated until it formed a clear solution, then it was allowed to cool to room temperature and left undisturbed for 24.0 h. Gel formation was confirmed by a vial inversion test. The results indicated that gelation was observed for N-BTA in alcohols such as methanol, ethanol, isopropanol, and *n*-butanol, but failed to form gels in aliphatic and apolar aromatic solvents (toluene, xylenes, and mesitylene), presumably due to their poor solubility (Table S1). We also tested the gelation abilities of N-BTA in high-polar solvents, but gels were not formed in these solvents. This prompted us to perform gelation studies in aqueous mixtures of high-polar solvents by introducing water as an anti-solvent because N-BTA is insoluble in water. Gelation was observed in an aqueous mixture (1:1, *v*/*v*) of high-polar solvents, such as DMF, DMSO, DMA, and DEA.

The minimum gelator concentration (MGC), which indicates the minimum quantity of gelator necessary for forming a stable gel network in a specific solvent, was evaluated by varying the quantity of the gelator (Table 1). The MGCs were similar for the aqueous mixtures of DMF, DMA, and DEA, but higher for DMSO. The MGCs for N-BTA in alcohols were similar but lower than those in aqueous mixtures, presumably due to the insolubility of N-BTA in the antisolvent.

### 2.3. Thermal Stability

The thermal stability of the N-BTA gels was evaluated by monitoring the transition temperature (*T_gel_*) at which the gel transformed into a liquid phase (gel–sol transition). All gels were prepared at the same concentration (4.0 wt/v%) in methanol, ethanol, isopropanol, *n*-butanol, and aqueous mixtures (1:1, *v*/*v*) of DMF, DMSO, DMA, and DEA in order to compare their thermal stabilities (Table 1). The gel–sol transition temperature in methanol was significantly lower than that of the gels in higher alcohols, indicating that increasing the chain length of the alcohols increased the stability of the gel network, leading to a significantly stronger network. The experiments performed with the aqueous mixtures (1:1, *v*/*v*) of DMF, DMA DEA, and DMSO indicated that the thermal stabilities of gels in DMSO/water (1:1, *v*/*v*) were slightly higher than the DMF/water (1:1, *v*/*v*) gels, but significantly higher compared to the other gels. The lower thermal stability of DMA/water (1:1, *v*/*v*) gel could be attributed to the better solubility of N-BTA in DMA.

### 2.4. Rheology

**Figure 1:**
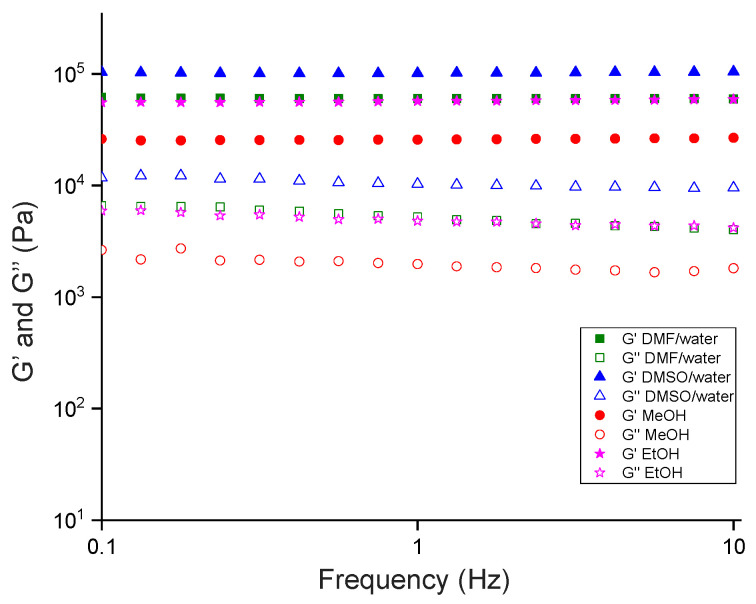
Frequency sweep experiments of gels in various solvents/solvent mixtures at 4.0 wt/v% at 20.0 °C with a constant strain of 0.01%.

Rheology is a valuable tool for studying the rigidity, deformation, and flow properties of gels, offering valuable insights into the underlying structural features of the gel network [46,47]. The mechanical strength of the gels was analyzed by performing amplitude and frequency-sweep experiments in methanol, ethanol, and aqueous mixtures (1:1, *v*/*v*) of DMF and DMSO at 4.0 wt/v%. Initially, a strain sweep was performed to identify the linear viscoelastic region (LVR), maintaining a constant frequency of 1.0 Hz, and the elastic modulus (G′) was independent of the applied strain. All the gels displayed a constant G′ up to 0.1% strain (Figures S1–S4). The point at which the elastic gel transforms into a viscous fluid, marked by a sudden decline in the G′, is referred to as the crossover point. Frequency-sweep experiments were conducted with a constant strain of 0.01% (within LVR) over a frequency range of 0.1–10.0 Hz, and the results displayed constant elastic (G′) and viscous (G″) moduli under varying frequencies. The comparison of mechanical strengths revealed that the gel in DMSO/water (1:1, *v*/*v*) exhibited higher storage modulus values compared to all other gels, suggesting a relatively stronger gel network (Figure 1).

### 2.5. Gel Morphology

**Figure 2:**
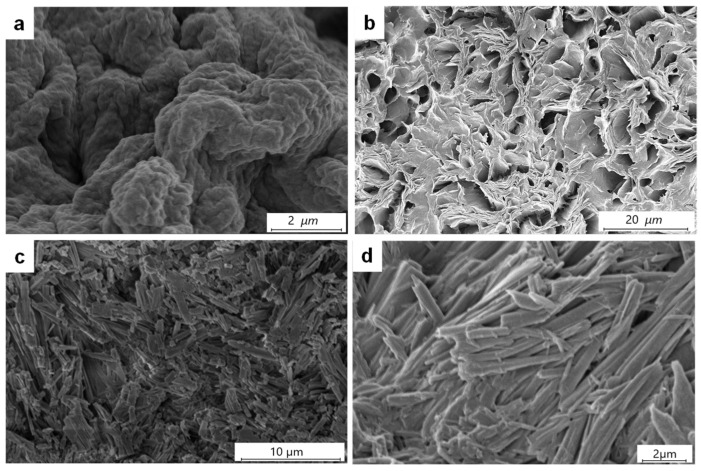
SEM images of N-BTA xerogels (2.0 wt/v%) from (**a**) methanol and (**b**) ethanol, and xerogels from (**c**) DMF/water and (**d**) DMSO/water (1:1, *v*/*v*) at 4.0 wt/v%, respectively.

The morphologies of the gel fibers can be visualized using modern microscopic techniques. Scanning electron microscopy (SEM) is one of the most useful techniques for elucidating gel fiber morphologies [48], which helps to evaluate the nanostructures of the LMWGs. The surface morphology of the fibrous network was analyzed by performing SEM on the dried gel samples, which were structurally similar to the actual gel network. However, artifacts of the drying process sometimes affect the structure; therefore, these measurements are not a perfect representation of the gel network [49]. The morphology of the gel network obtained by drying the gels of N-BTA from methanol (2.0 wt/v%) displayed zoetic features (Figure 2a), but flake-like morphologies with fiber dimensions ranging from 0.1 to 1.0 µm (Figure 2b) were observed for ethanol xerogels.

Similar morphologies were observed for isopropanol and *n*-butanol xerogels at 2.0 wt/v% (Figure S5). The xerogels obtained from the aqueous mixtures (1.1, *v*/*v*) of DMF and DMSO at 4.0 wt/v% displayed needle-shaped morphologies (Figure 2c,d) with thicknesses ranging from 0.2 to 1.5 µm.

### 2.6. Powder X-ray Powder Diffraction (PXRD)

**Figure 3:**
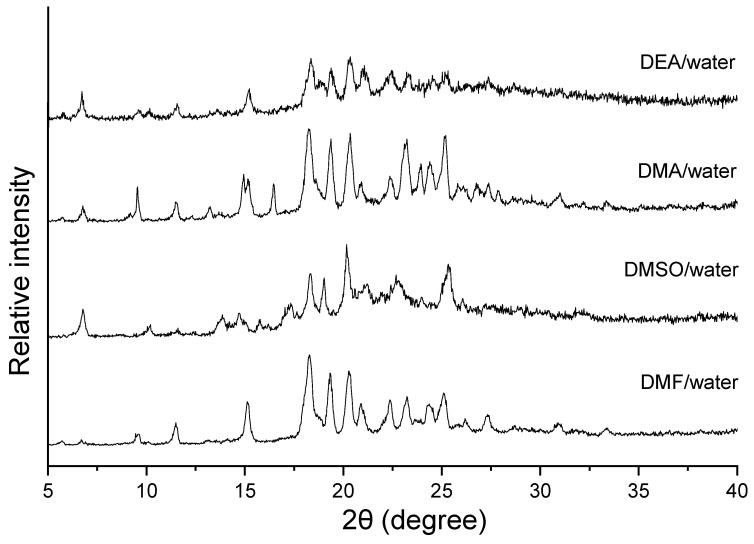
PXRD patterns of the dried gels (4.0 wt/v%) from the aqueous solutions of DMF, DMSO, DEA, and DMA (1:1, *v*/*v*).

Powder X-ray diffraction (PXRD) is an important tool for confirming the phase purity of a material. PXRD can be used to analyze the molecular packing and the self-assembly process of LMWGs [35,48,50,51,52]. Our group showed that comparing the PXRD pattern of the xerogels with the simulated pattern of the gelator structure could yield valuable insights into the fundamental interactions within the gel network architecture [35,50,51,52]. This was a promising approach, but sometimes the drying process led to morphological changes or the phase transition of the fibrous networks [49]. We used PXRD to evaluate the self-assembly modes of N-BTA in various solvents by comparing the PXRD patterns of the xerogels for the corresponding solvents. The comparison of the PXRD patterns of the xerogels (4.0 wt/v%) taken from the aqueous solutions of DMF, DMSO, DEA, and DMA revealed that the patterns were superimposable (Figure 3).

However, the PXRD patterns obtained from the xerogels from the alcohols displayed broad peaks, presumably due to poor crystallinity of the gelators in these solvents, but most of the peaks matched with the PXRD patterns obtained for the aqueous mixtures (Figure S6) and the bulk material. These results indicate that a similar gel network for N-BTA xerogels was observed, irrespective of the solvents used.

### 2.7. Stimuli-Responsive Properties

**Figure 4:**
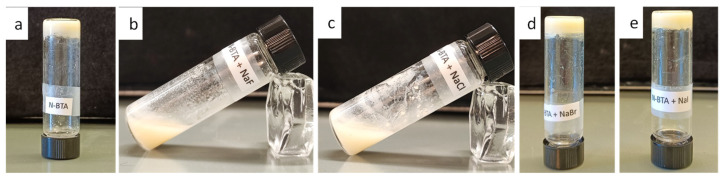
Stimuli-responsive properties of the (**a**) N-BTA gels in DMSO/water mixture (1:1, *v*/*v*) with sodium halides (1.0 equiv.), such as (**b**) fluoride, (**c**) chloride, (**d**) bromide, and (**e**) iodide salts.

Supramolecular gels are an excellent class of stimuli-responsive materials, as their gelation process can be modulated by various external stimuli like pH, light, sound, and redox reactions, as well as by the introduction of external factors such as salts or ions [15,16,17,18,19]. The interactions between cation/anions and gelator molecules can be either constructive or destructive, depending on the electrostatic interaction and acidic/basic characteristics of the cations/anions [15,53]. A positive interaction has the potential to start or enhance the process of gelation, whereas a negative interaction might result in the dissolution or collapse of the gel network [15,16,17,18,19]. LMWGs based on amide moieties are promising candidates for anions sensing [15,16,17,18,19,54], and we have reported the anion-sensing capabilities of amide-based gelators [55,56]. The presence of amide moieties in the gelators prompted us to evaluate the anion-sensing abilities of the N-BTA gelator. The stimuli-responsive properties of the gels were analyzed by treating the gels with various anions of sodium and potassium salts. The gels were prepared at MGC (3.6 wt/v%) using sodium/potassium (1.0 equiv.) halides, nitrate, sulphate, acetate, and cyanide ions in a DMSO/water mixture (1:1, *v*/*v*), and the gel state properties were compared to those of the native gelator. The fluoride, chloride and cyanide salts disrupted the gel network, but the gel network remained intact in the presence of nitrate, sulphate, acetate, bromide, and iodide salts (Figure 4 and Figure S7). The ability of anions to induce gelation was tested by performing gelation below MGC (3.0 wt/v%) and gel formation in the presence of nitrate, sulphate, acetate, bromide, and iodide salts (1.0 equiv.) confirmed the occurrence of anion-induced gelation (Figure S8).

**Figure 5:**
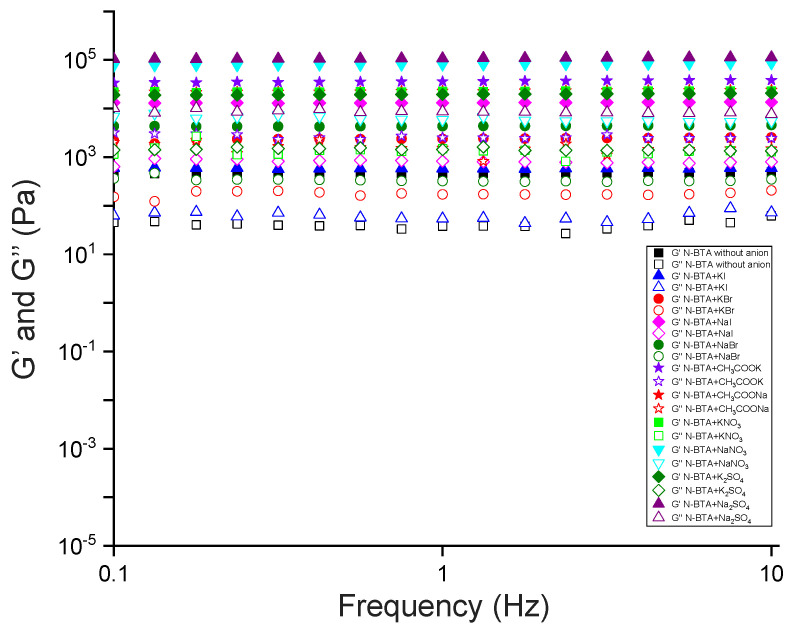
Frequency sweep experiments of N-BTA gels (3.6 wt/v%) in the presence of various sodium/potassium salts of bromide, iodide, nitrate, acetate, and sulphate.

These results indicate that smaller and more nucleophilic anions, such as cyanide, fluoride, and chloride ions, disrupt hydrogen bonding networks (destructive interaction), but larger and less nucleophilic anions such as bromide, iodide, nitrate, sulphate and acetate enhance (constructive interaction) the formation of gel networks. The stimuli-responsive nature (ON/OFF gelation) of the N-BTA gelator towards anions indicates that the size of the anion is crucial for ensuring the effective interaction of the gelator with the anion [57,58,59]. Thus, N-BTA compound can be considered to be size-selective anion-responsive supramolecular gels, which can detect cyanide, fluoride and chloride ions in aqueous solution by monitoring the gel-to-sol transition. The mechanical and thermal strength of these gels was evaluated in order to observe the effect of anions on the stability of gel networks (Figure 5 and Table S2), and the results indicated that the presence of anions enhanced the thermal and mechanical stability of the LMWGs.

### 2.8. Dye Adsorption Studies

For the dye adsorption studies, we chose methylene blue (MB), a widely recognized cationic dye that is extensively utilized in the textile, clothing, paper, pharmaceutical, cosmetics, and leather industries [60]. Furthermore, its limited biodegradability and its mutagenic and carcinogenic properties make the water purification process challenging. Water contamination by MB is a threat to both human health and plant life. In humans, exposure to the dye can result in a range of afflictions, including vomiting, jaundice, cyanosis, and increased heart rates. Similarly, the presence of MB causes growth inhibition and the reduction of pigments in plants [60]. We also chose methyl orange (MO), an anionic azo dye found in effluents from the textile, printing, culinary, pharmaceutical, and paper industries, as well as research labs [61]. The presence of MO and its degradation products is highly carcinogenic and toxic to land and aquatic environments [62].

**Figure 6:**
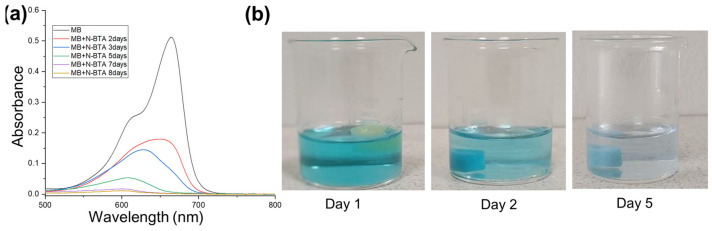
(**a**) UV-vis experiments of MB (7.5 × 10^−6^ M) in deionized water and (**b**) upon adding the N-BTA gel prepared in ethanol (2.0 wt/v%) to MB (7.5 × 10^−6^ M).

The concentration of the dyes in water was optimized by performing the UV-vis experiments and the maximum absorbance of methylene blue (7.5 × 10^−6^ M) was 665 nm (Figure 6a). The gels prepared from ethanol (2.0 wt/v%) were loaded carefully into a solution of the MB (10.0 mL) in a 25.0 mL beaker, and the absorbance was recorded over 8 days at pH 4.2 and 21.0 °C (Figure 6a). We observed a decrease in absorbance with MB in the solution after a span of 2 days. The blue-colored MB solution turned colorless after 5 days (Figure 6b), and the absorbance peaks almost disappeared after 7 days. The adsorbed amount of MB was calculated using UV-vis absorption and we observed that the concentration of MB gradually decreased over 8 days, with an adsorption rate of 97.9% (Figure S9).

**Figure 7:**
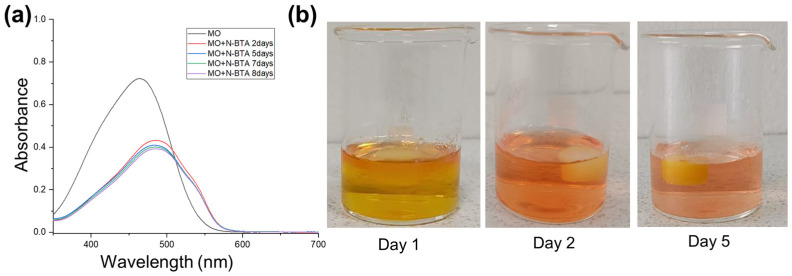
UV-vis: (**a**) UV-vis experiments of MO (5.0 × 10^−5^ M) in deionized water and (**b**) upon adding the N-BTA gel prepared in ethanol (2.0 wt/v%) to MO (5.0 × 10^−5^ M).

The UV-vis experiments performed at an optimized concentration of MO (5.0 × 10^−5^ M) in water at pH 3.5 and 21.0 °C revealed the maximum absorbance was 465 nm (Figure 7a). The procedure for the dye adsorption process was similar to that for MB adsorption. The experiments showed a decrease in absorbance after 2 days with a color change from yellow to orange over time and a shift in absorbance maxima towards higher wavelengths. The color change and the shift in absorbance might have been due to the change in pH from 5.0 (MO solution) to 3.5 after adding N-BTA gels to the MO solution [63]. After 5 days, the absorbance was further reduced (Figure 7a,b), and around 60.0% of the dye was removed within 8 days (Figure S10). The dye adsorption of N-BTA was confirmed by a series of experiments with N-BTA and the dyes under various conditions. Initially, dry N-BTA (5.0 mg) was dispersed in an aqueous solution of MB and MO, and no color change was observed for MB. However, the yellow color was transformed into orange in the case of MO, which was similar to what occurred in the gel state experiments (Figure S11). We repeated the MO dye adsorption experiments with N-BTA gels in different solvents such as isopropanol, *n*-butanol, and DMSO/water, and similar color changes/shifts in absorbance maxima were observed (Figure S12). We were unable to perform the re-adsorption studies with N-BTA gels because the recovered gels from both dyes were not reusable.

The adsorption capacity (*Q_t_*) and adsorption efficiency (*R_e_*) of N-BTA when used on the dye were calculated (Table 2) from the concentration of dyes before and after adsorption using the following equations [23]:

$$Q_{t}=\frac{V\left(C_{0}-C_{t}\right)}{m}\;\left(\mathrm{mg}/g\right)$$

$$R_{e}=\frac{\left(C_{0}-C_{t}\right)}{C_{0}}\times 100\%$$

where *C*_0_ is the initial dye concentration in solution (mg/L), *C_t_* is the residual dye concentration in solution at time *t* (mg/L), *V* is the solution volume (L), and *m* is the mass of the dry adsorbent (g).

**Figure 8:**
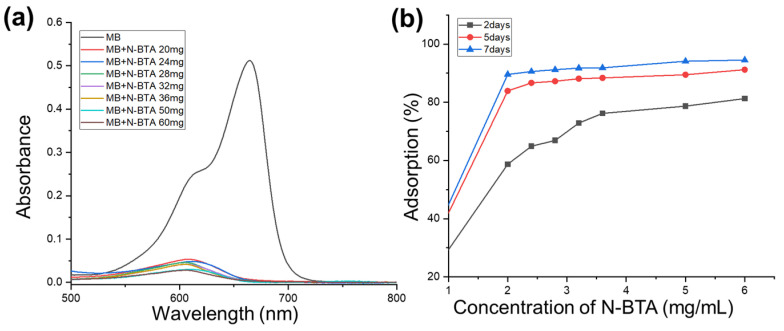
(**a**) UV-vis experiments of MB (7.5 × 10^−5^ M) in deionized water with varying concentrations of N-BTA after 7 days and (**b**) adsorption ratio of MB with varying concentrations of N-BTA after 2, 5, and 7 days.

The adsorption efficiency with respect to the concentration of the gelator was studied by varying the quantity of N-BTA from 20.0 to 60.0 mg. The N-BTA gels were immersed in 10.0 mL of dye solutions, the absorbance was recorded over a span of 2, 5, and 7 days, and we calculated the adsorption efficiency (Figure 8 and Figures S13–S18). The results showed that adsorption efficiency increased with the concentration of N-BTA and reached a plateau (Figure 8b). The adsorption ratio of MB remained at approximately 94.5% when the concentration of N-BTA was 6.0 mg/mL. The adsorption ratio of MO was approximately 76.5% when the concentration of N-BTA was 6.0 mg/mL (Figure S18). These experiments reveal the ability of the N-BTA gel to adsorb MB and MO dyes from water.

Our adsorption studies indicate that N-BTA gel is more efficient in terms of removing cationic methylene blue dye than the anionic methyl orange. These results demonstrate that the *C_3_*-symmetric BTA (N-BTA) LMWGs can interact with both cationic and anionic dyes in water, which reveals the versatile properties of N-BTA gel as a selective anion sensor and dye adsorber. As mentioned in the introduction, synthesizing N-BTA molecules is challenging, and we have shown that simple alteration of the functional group will lead to *C_3_*-symmetric compounds with intriguing properties.

## 3. Materials and Methods

The starting materials and solvents were purchased commercially from Sigma-Aldrich (MEDOR ehf, Reykjavik, Iceland), Fluorochem UK, and TCI-Europe (Boereveldseweg, Belgium) and were utilized as supplied. Deionized water was used for the gelation tests. We characterized the molecules using ^1^H and ^13^C NMR spectra (Figures S19–S22), which were recorded in a Bruker Avance 400 spectrometer (Rheinstetten, Germany), and the SEM images (Carl Zeiss, Oberkochen, Germany) were recorded on a Leo Supra 25 microscope. The rheological experiments were performed on an Anton Paar modular compact rheometer MCR 302 (Graz, Austria). Powder X-ray diffraction (PXRD) experiments were performed with bulk compounds and xerogel using a PANalytical instrument (Almelo, The Netherlands).

### 3.1. Synthesis of Ligands

We synthesized the 1,3,5-tris(azidomethyl)benzene by modifying the reported procedure for obtaining a similar reaction [64].

#### 3.1.1. Synthesis of Benzene-1,3,5-Triyltrimethanamine

The corresponding azide (2.7 g, 11.1 mmol) was dissolved in a mixture of 60.0 mL of THF and 7.5 mL of water. This solution was treated with triphenylphosphine (14.6 g, 55.5 mmol) and then stirred at room temperature for 15 h. The completion of the reaction was monitored using a TLC, and the mixture was concentrated to ∼10.0 mL. The mixture was treated with 2.0 M HCl (20.0 mL), resulting in a hydrochloride salt of the amine, which was washed with DCM (3 × 30 mL) to remove the other organic impurities. The aqueous layer was evaporated in a fume hood to obtain a white powder, which was washed with DCM to yield the amine hydrochloride. Yield: 2.89 g, 95.0%. ^1^H NMR (400 MHz, DMSO-*d_6_*) δ (ppm): 8.65 (s, 9H), 7.66 (s, 3H), and 4.01 (s, 6H). ^13^C {^1^H} NMR (100 MHz, DMSO-*d_6_*) δ (ppm): 134.61, 129.68, and 42.06. MS (ESI): calcd for C_9_H_15_N_3_Na [M + Na]^+^, 188.1158; found, 188.1146.

#### 3.1.2. Synthesis of Triethyl 4,4′,4″-(((benzene-1,3,5-triyltris(methylene))tris(azanediyl))tris(carbonyl))tribenzoate (N-BTA)

The benzene-1,3,5-triyltrimethanamine hydrochloride (0.89 g, 3.2 mmol) sample was dissolved in 70.0 mL of THF by adding triethylamine (3.1 mL, 22.6 mmol) under a nitrogen atmosphere and the product was kept in an ice bath. The acid chloride of 4-(ethoxycarbonyl) benzoic acid (2.7 g, 11.1 mmol) was then added dropwise after being dissolved in 50.0 mL of THF and stirred at room temperature for an hour. The mixture was refluxed overnight, cooled, and evaporated to dryness. The crude mixture was kept in the fume hood for a day and then stirred with 5.0% sodium bicarbonate for 12.0 h. The mixture was filtered, washed with copious amounts of water, and dried to obtain the corresponding amide as a white powder. The product was then recrystallized in ethanol. Yield: 1.6 g, 71.5%. ^1^H NMR (400 MHz, DMSO-*d_6_*) δ (ppm): 9.22 (t, J = 6.1 Hz, 3H), 7.93 (q, J = 8.7 Hz, 12H), 7.17 (s, 3H), 4.47 (d, J = 6.0 Hz, 6H), 4.34 (q, J = 7.1 Hz, 6H), 1.34 (t, J = 7.1 Hz, 9H). ^13^C {^1^H} NMR (100 MHz, DMSO-*d_6_*) δ (ppm): 170.63, 170.37, 144.90, 143.59, 137.21, 134.23, 132.75, 129.55, 66.25, 47.75, 19.32. MS (ESI): calcd for C_39_H_39_N_3_O_9_Na [M + Na]^+^, 716.2579; found, 716.2564.

### 3.2. Gelation Studies

We added 10.0 mg of N-BTA and 1.0 mL of the solvent to a standard 7.0 mL vial (ID = 15.0 mm), and the vial was sealed. The mixture was sonicated and heated gradually to produce a transparent solution, and the mixture was kept undisturbed for 24.0 h. A gelation confirmation test was performed using an inversion test. Gelation experiments were also performed in a 1:1, *v*/*v* mixed aqueous system, where 10.0 mg of the N-BTA was dissolved in 0.5 mL of the suitable solvent in a standard 7.0 mL vial. Subsequently, 0.5 mL of deionized water was added to the solution. The vial was sealed, and the solution was subjected to sonication and heated slowly to achieve a clear solution. The solution was kept undisturbed, and gel formation was confirmed using a vial inversion test. The experiments were replicated, using higher doses of the compound (up to 50.0 mg) to check gel formation at higher concentrations.

#### 3.2.1. Minimum Gelator Concentration (MGC)

The MGC experiment was conducted using appropriate solvents, where different quantities of the compounds were weighed in a standard 7.0 mL vial. Then, 1.0 mL of solvent or solvent mixture was added. The vial was sealed, sonicated, and heated gradually to achieve complete dissolution. Subsequently, the solution was maintained at room temperature to facilitate gel formation. The minimum quantity of the compound necessary for the formation of a stable gel within a 24.0 h period was determined as the MGC.

#### 3.2.2. *T_gel_* Experiments

The necessary quantity of gelator and 1.0 mL of solvent were added to a 7.0 mL standard vial. The solution was subjected to sonication and heated slowly until it dissolved, and the product was then allowed to gel without any disturbance. Following 24.0 h, a ball-drop method was employed to observe the gel-to-sol transition temperature (*T_gel_*). A spherical-shaped glass ball was positioned on the gel surface and the vials were sealed before being submerged in an oil bath. The oil bath equipped with a magnetic stirrer was gradually heated at a rate of 10.0 °C per minute, and a thermometer was utilized to monitor the temperature of the oil bath. As the ambient temperature was reached, the glass ball gradually became submerged within the gels, and the temperature at which the ball made contact with the lower surface of the vial was recorded as *T_gel_*. The experiments were repeated three times, and the average was taken to be the *T_gel_*.

### 3.3. Rheology

Rheological measurements were conducted using an Anton Paar Modular Compact Rheometer MCR 302 and the experiments were repeated three times for each sample. Mechanical strength was measured using a 2.5 cm stainless-steel parallel-plate geometry setup. In all instances, oscillatory measurements were performed under a constant temperature of 20.0 °C. A Peltier temperature control hood was used as a solvent trap, which maintained a temperature of 20.0 °C for frequency and amplitude sweeps. A suitable quantity of the respective gelator was dissolved in 1.0 mL of solvent to make the gels. After 24 h, the studies were conducted by transferring the gel onto the plate. A consistent frequency of 1.0 Hz was preserved during the amplitude sweep, while the logarithmic ramp strain (Y) ranged between 0.01 and 100%. The frequency sweep was conducted inside the linear viscoelasticity domain (0.01% strain) between 0.1 and 10.0 Hz.

### 3.4. Scanning Electron Microscopy (SEM)

A Leo Supra 25 microscope was used to perform the SEM imaging to analyze the surface morphologies of the xerogels. Gels of the compound were prepared in methanol, ethanol, isopropanol, and *n*-butanol at 2.0 wt/v%, and in aqueous mixtures (1:1, *v*/*v*) of DMF and DMSO at 4.0 wt/v%. The gels were filtered after 24 h and dried under a fume hood to obtain xerogels. A small amount of xerogel was put on a pin mount with the carbon tab on top. It was subsequently coated with gold for 5–6 min (12.0 nm thickness) to keep the surface from charging and was then loaded. mages were taken at a working distance of 3.0–4.0 mm and an operating voltage of 3.0 kV. The SEM picture was taken with an in-lens detector.

### 3.5. Powder X-ray Diffraction

We obtained samples of microcrystalline material of N-BTA in ethanol (20.0 mg in 2.0 mL). The crystalline material was filtered, dried in air, and ground down to a fine powder. The xerogels of the compound were prepared from corresponding gels made from methanol, ethanol, isopropanol, and n-butanol, and from aqueous mixtures of DMSO, DMF, DMA, and DEA at 4.0 wt/v%. Gels were further filtered and dried in a fume hood to obtain xerogels. All the tests were conducted using a PANalytical instrument with a Cu anode (Almelo, The Netherlands), with a step size of 0.025 and a range of 2θ, running from 4.0 to 50.0.

### 3.6. Stimuli-Responsive Properties

We dissolved 36.0 mg of N-BTA (MGC) in 0.5 mL of DMSO and dissolved sodium/potassium halides, nitrate, carbonate, cyanide, sulphate, and acetate (1.0 equiv.) in 0.5 mL water. The solutions were mixed, heated until they dissolved, and left undisturbed for gel formation. We performed thermal analysis of the obtained gels using T_gel_ studies, and the mechanical properties were recorded using rheological measurements (frequency sweep) following the procedures described in Section 3.2.2 and Section 3.3, respectively, and the experiments were repeated two times for each anion.

### 3.7. UV–Visible Spectroscopy

UV-vis absorptions were recorded on the Agilent Cary UV-vis Multicell Peltier spectrometer. The MB and MO dye solutions were prepared in water (10.0 mL) at a concentration of 7.5 × 10^−6^ M and 5.0 × 10^−5^ M via dilution from a higher known concentration. In the dye adsorption experiment, data was collected with a bandwidth of 2.0 nm. Initially, we prepared the solution of the dye in water in a 25.0 mL beaker at the required concentration, and then the absorbance was recorded. We then carefully immersed the gel in a dye solution, sealed the beaker, and recorded the absorbance of the solution after 2 to 8 days.

#### 3.7.1. Dye Adsorption Studies with Varying Concentrations of N-BTA

Gels of the compound were prepared at various concentrations (wt/v%) of N-BTA (2.0, 2.4, 2.8, 3.2, 3.6, 5.0 and 6.0) in ethanol and immersed in 10.0 mL aqueous solutions of MB (7.5 × 10^−6^ M) and MO (5.0 × 10^−5^ M). The absorbance was recorded after 2, 5, and 7 days.

#### 3.7.2. Dye Adsorption Studies of MO with N-BTA Gel from Other Solvents

Gels of the compound were prepared at 4.0 wt/v% in *n*-butanol, isopropanol, and DMSO/water mixture (1:1, *v*/*v*) and immersed in an aqueous solution (10.0 mL) of MO. The absorbance was recorded after 2 days.

## 4. Conclusions

We successfully synthesized *C_3_*-symmetric N-centered BTA (N-BTA) by modifying BTA molecules with flexible linkers. Our gelation studies revealed that N-BTA formed gels in alcohols and aqueous mixtures of high-polar solvents. We characterized the gels using standard gelation techniques and analyzed their morphologies using SEM. The comparison of mechanical strength in different solvents revealed that the gel from DMSO/water (1:1, *v*/*v*) mixture displayed a stronger gel network, which correlated well with the thermal stability experiments. The xerogels from aqueous mixtures displayed needle-shaped morphologies, whereas the xerogels from alcohols displayed zoetic and flake-like morphologies. The comparison of the PXRD patterns of the xerogels obtained from the aqueous mixtures found that they perfectly matched, suggesting a similar mode of packing. The xerogels from the alcohols were less crystalline, displaying broad peaks, but most of the peaks matched with those of the aqueous mixtures and bulk material. The anion-sensing properties of N-BTA were evaluated in the presence of sodium and potassium salts in a DMSO/water mixture (1:1, *v*/*v*) at MGC and below. The gel network was disrupted by cyanide, chloride and fluoride salts, but remained intact in the presence of nitrate, sulphate, acetate, bromide and iodide salts. The experiments performed below MGC revealed that nitrate, sulphate, acetate, bromide and iodide salts induced gelation. Analysis of the mechanical and thermal strengths of these gels in comparison with the native gelator revealed that the N-BTA gels exhibited improved thermal and mechanical stability in the presence of nitrate, sulphate, acetate, bromide and iodide salts. The dye adsorption capability of N-BTA was analyzed by dispersing the ethanolic gel in an aqueous solution of MB and MO, resulting in a colorless solution of MB after 5 days. The UV-vis absorption studies revealed a reduction in absorbance for MB within 2 days, which was mostly absorbed within 8 days. Similarly, the MO solution also showed a decrease in absorbance after 8 days, but the adsorption of MO from water by the N-BTA gel was lower compared to that of the cationic MB dye. In summary, this study highlights the potential of *C_3_*-symmetric N-BTA for application in anion-sensing and dye removal from wastewater treatment. This study demonstrates an easy synthetic route for synthesizing N-centered BTA molecules with intriguing properties by altering functional groups.

## Figures and Tables

**Table 1:** Minimum Gelator Concentration (MGC) and *T_gel_* (4.0 wt/v%) of the gelator.

Solvent	MGC (wt/v%)	*T_gel_* (°C)
Methanol	1.9	79.9
Ethanol	1.9	82.9
Isopropanol	2.0	94.3
*n*-butanol	2.0	96.1
DMF/water (1:1, *v*/*v*)	2.8	92.8
DMSO/water (1:1, *v*/*v*)	3.6	99.1
DMA/water (1:1, *v*/*v*)	2.5	58.3
DEA/water (1:1, *v*/*v*)	2.7	83.9

**Table 2:** Comparison of adsorption capacity (*Q_t_*) and adsorption efficiency (*R_e_*) of N-BTA when used on MB and MO.

Day	Adsorption Capacity (mg/g)		Adsorption Efficiency (%)	
	MB	MO	MB	MO
2	0.78	4.92	64.94	60.11
5	1.07	5.09	89.58	62.19
7	1.15	5.14	96.64	62.81
8	1.17	5.21	97.87	63.61

## Data Availability

All the details are given in the Supplementary Materials.

## Supplementary Materials

The following supporting information can be downloaded at: https://www.mdpi.com/article/10.3390/molecules29092149/s1, Figure S1. Amplitude sweep measurement was performed for the N-BTA gel in EtOH at 4.0 wt/v%; Figure S2. Amplitude sweep measurement was performed for the N-BTA gel in MeOH at 4.0 wt/v%; Figure S3. Amplitude sweep measurement was performed for the N-BTA gel in DMSO/water at 4.0 wt/v%: Figure S4. Amplitude sweep measurement was performed for the N-BTA gel in DMF/water at 4.0 wt/v%; Figure S5. SEM images of N-BTA xerogels (2.0 wt/v%) in (a) isopropanol and (b) *n*-butanol; Figure S6. Comparison of PXRD patterns of the dried gels (4.0 wt/v%) from alcohols, DMF/water, and bulk material; Figure S7. Stimuli-responsive properties of the N-BTA gels with various anions (1.0 eq) at MGC in DMSO/water (1:1, *v*/*v*); (a) potassium halides, (b) sodium and (c) potassium salts of cyanide, sulphate, nitrate and acetate anions, respectively; Figure S8. (a) N-BTA gels below MGC in DMSO/water (1:1, *v*/*v*) and anion induced gelation with (b) sodium and (c) potassium salts such as bromide, iodide, nitrate, sulphate and acetate ions (1.0 eq.); Figure S9. The time-dependent adsorption of MB by N-BTA gel; Figure S10. The time-dependent adsorption of MO by N-BTA gel; Figure S11. Aqueous solution of (a) MB and MO before and after the addition of dry N-BTA; Figure S12. UV-vis experiments of MO (5.0 × 10^−5^ M) with N-BTA gel from n-butanol, isopropanol, and DMSO/water; Figure S13. UV-vis experiments of MB (7.5. × 10^−6^ M) with different concentrations of N-BTA after 2 days; Figure S14. UV-vis experiments of MB (7.5. × 10^−6^ M) with different concentrations of N-BTA after 5 days; Figure S15. UV-vis experiments of MO (5.0 × 10^−5^ M) with different concentrations of N-BTA after 2 days; Figure S16. UV-vis experiments of MO (5.0 × 10^−5^ M) with different concentrations of N-BTA after 5 days; Figure S17. UV-vis experiments of MO (5.0 × 10^−5^ M) with different concentrations of N-BTA after 7 days; Figure S18. Adsorption ratio of MO with varying concentrations of N-BTA after 2, 5, and 7 days; Figure S19. ^1^H NMR spectrum of benzene-1,3,5-triyltrimethanamine; Figure S20. ^13^C NMR spectrum of benzene-1,3,5-triyltrimethanamine, Figure S21; ^1^H NMR spectrum of N-BTA; Figure S22. ^13^C NMR spectrum of N-BTA; Table S1. Gelation Experiments; Table S2: T_gel_ studies with the gels in DMSO/water (1:1, *v*/*v*) in the presence potassium and sodium salts (1.0 equiv.).
