# Supplementary material for: Supramolecular Gels Based on C3-Symmetric Amides: Application in Anion-Sensing and Removal of Dyes from Water
Source: Molecules. 2024 May 5;29(9):2149. doi: 10.3390/molecules29092149 (PMC11085098; doi:10.3390/molecules29092149)
Supplement: Supplementary file 1 [file molecules-29-02149-s001.zip › molecules-2966975-supplementary.pdf]

SUPPORTING INFORMATION

Supramolecular Gels Based on C3-Symmetric Amides: Application in Anion-Sensing and Removal of Dyes from Water

Geethanjali Kuppadakkath, Sreejith Sudhakaran Jayabhavan, and Krishna K. Damodaran*

Contents

1. Gelation studies………………………………………………………………………………… 2
2. Rheology ………………………………………………………………………………………… 3
3. Scanning electron microscopy………………………………………………………… 5
4. Powder X-ray diffraction…………………………………………………………………… 6
5. Anion sensing……………………………………………………………………………… 7
6. Dye Adsorption studies………………………………………………………………… 9
7. NMR spectra ………………………………………………………………………………… 14

1. Gelation studies

Table S1. Gelation Experiments

Solvent	Gelation test at 1.0 wt/v%
DMF/water (1:1, v/v)	G**
DMSO/water (1:1, v/v)	G$
DMA/water (1:1, v/v)	G**
DEF/water (1:1, v/v)	G**
DEA/water (1:1, v/v)	G**
p-xylene	I
o-xylene	I
m-xylene	I
mesitylene	I
toluene	I
ethanol	G*
methanol	G*
isopropanol	G*
n-butanol	G*
acetonitrile	I
ethyl acetate	I
dichloromethane	I
chloroform	I
hexane	I
acetone	I

G= gel, I= insoluble, G*= 2.0 wt/v%, G**= 3.0 wt/v%, G$= 4.0 wt/v%.

2. Rheology

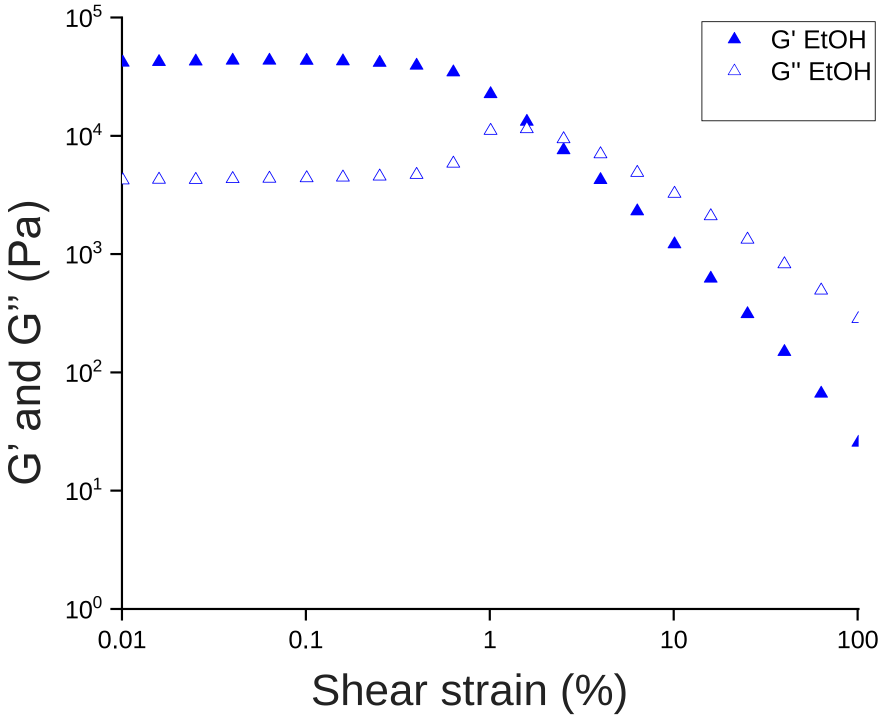

Figure S1. Amplitude sweep measurement was performed for the N-BTA gel in EtOH at 4.0 wt/v%.

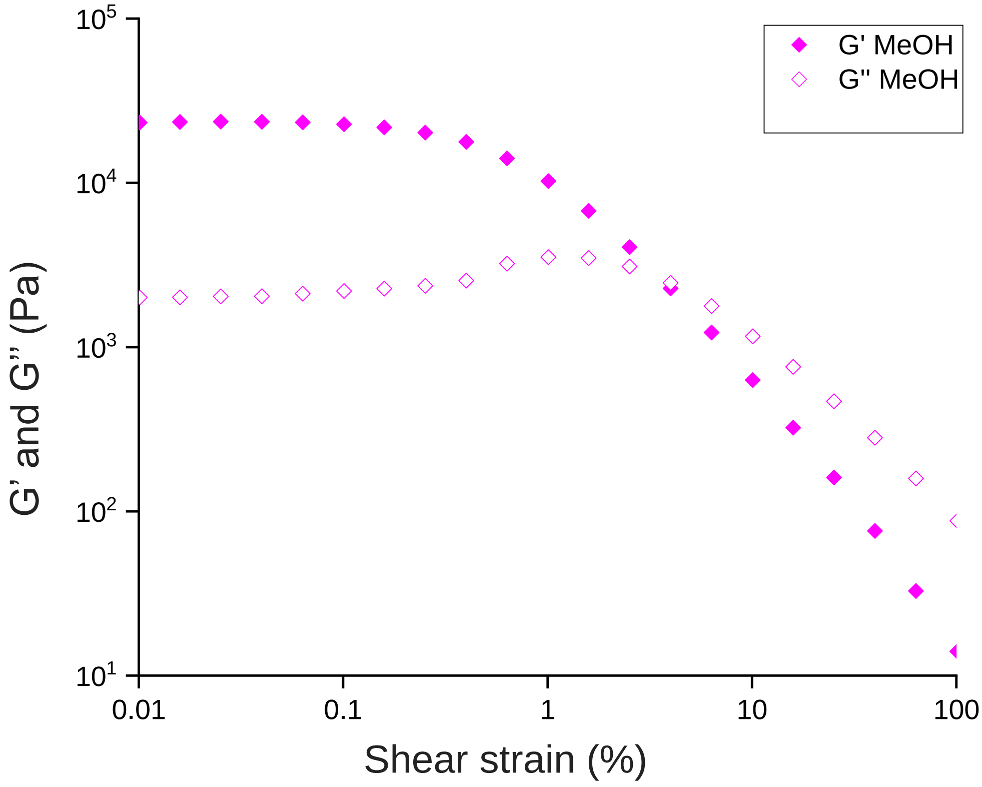

Figure S2. Amplitude sweep measurement was performed for the N-BTA gel in MeOH at 4.0 wt/v%.

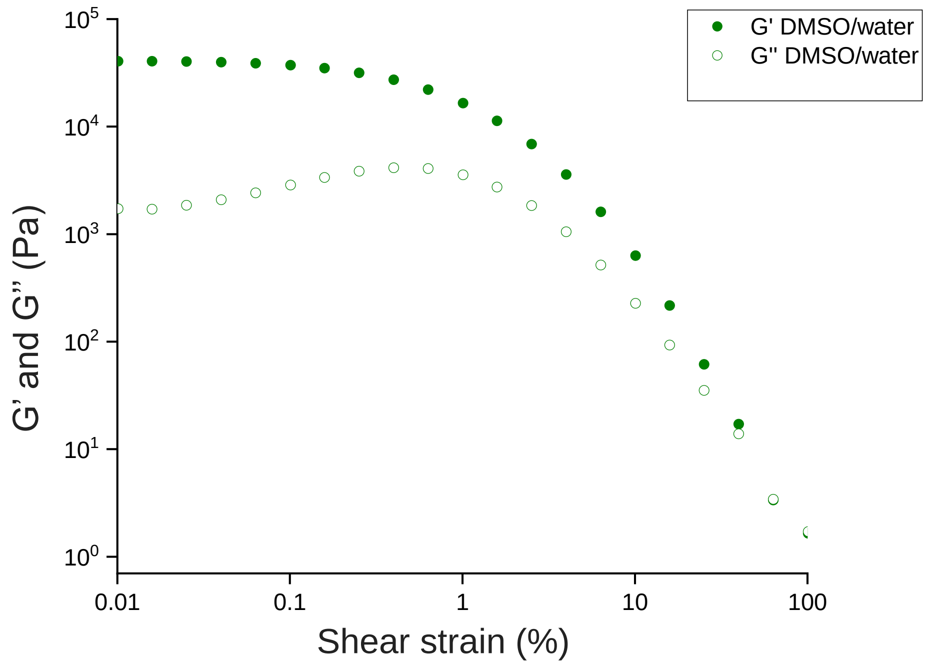

Figure S3. Amplitude sweep measurement was performed for the N-BTA gel in DMSO/water at 4.0 wt/v%.

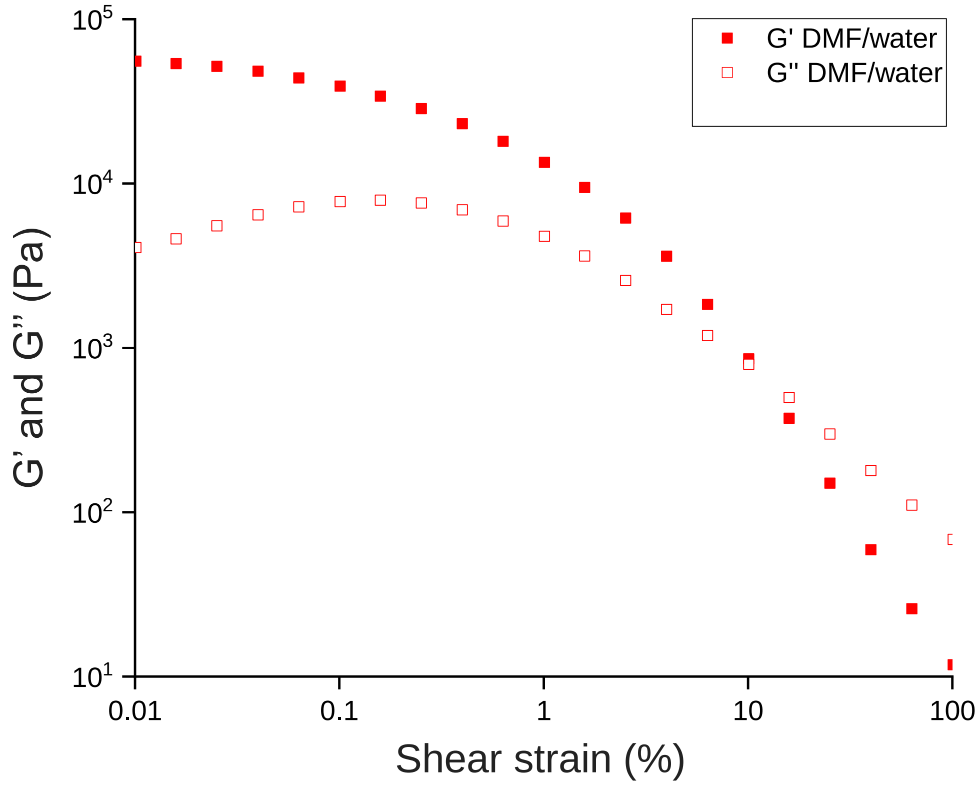

Figure S4. Amplitude sweep measurement was performed for the N-BTA gel in DMF/water at 4.0 wt/v%.

3. Scanning Electron Microscopy (SEM)

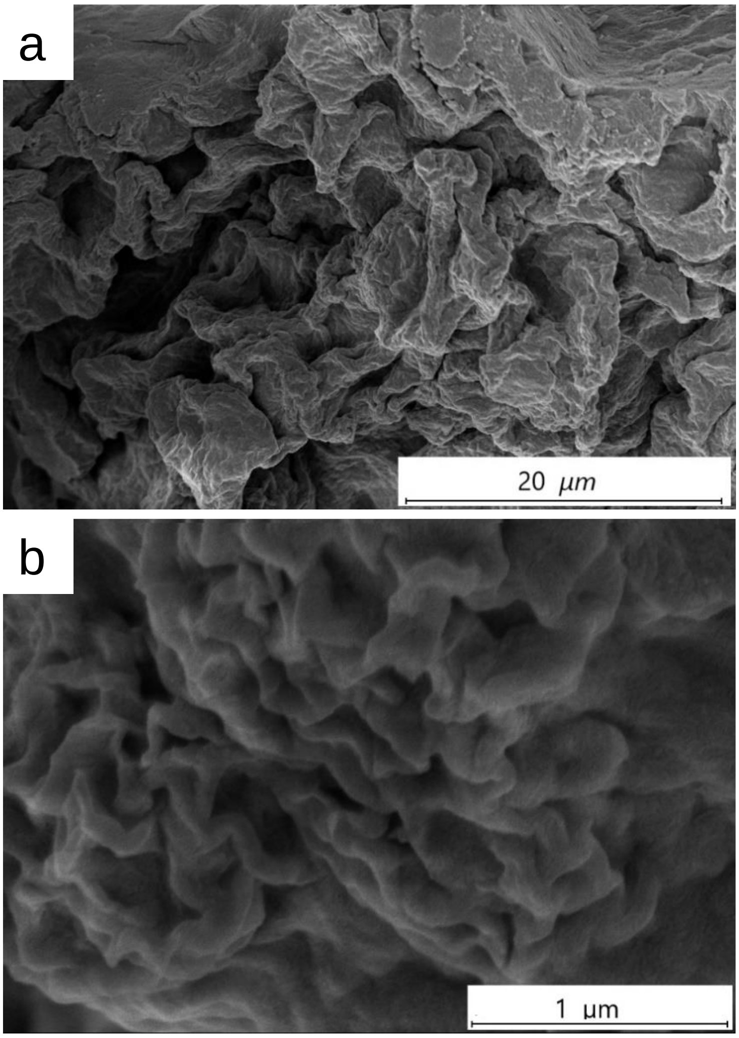

Figure S5. SEM images of N-BTA xerogels (2.0 wt/v%) in (a) isopropanol and (b) *n*-butanol.

4. Powder X-ray diffraction

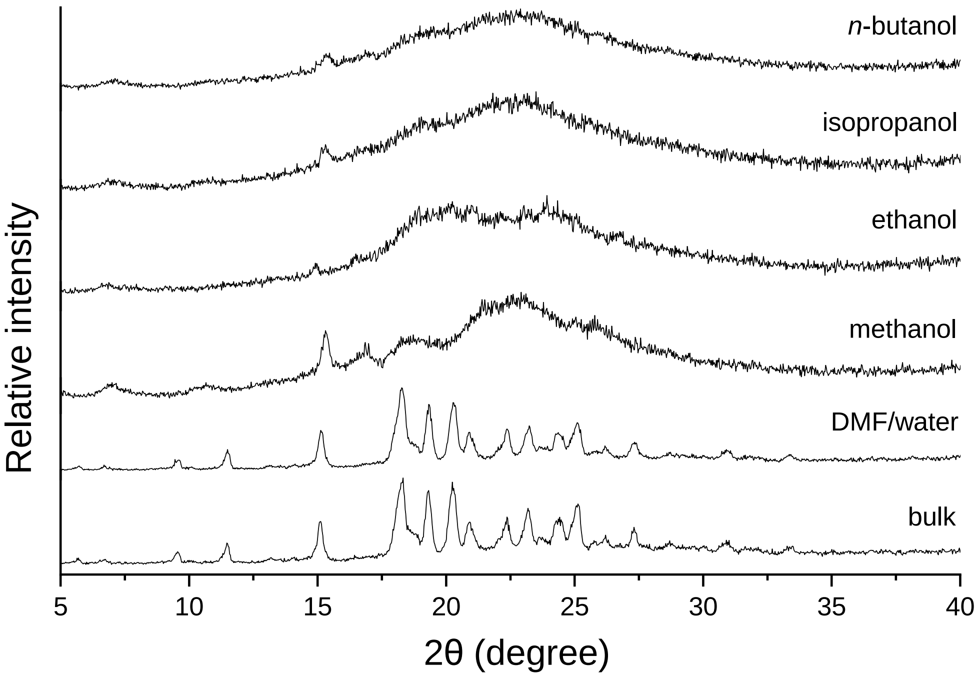

Figure S6. Comparison of PXRD patterns of the dried gels (4.0 wt/v%) from alcohols, DMF/water, and bulk material.

5. Anion sensing

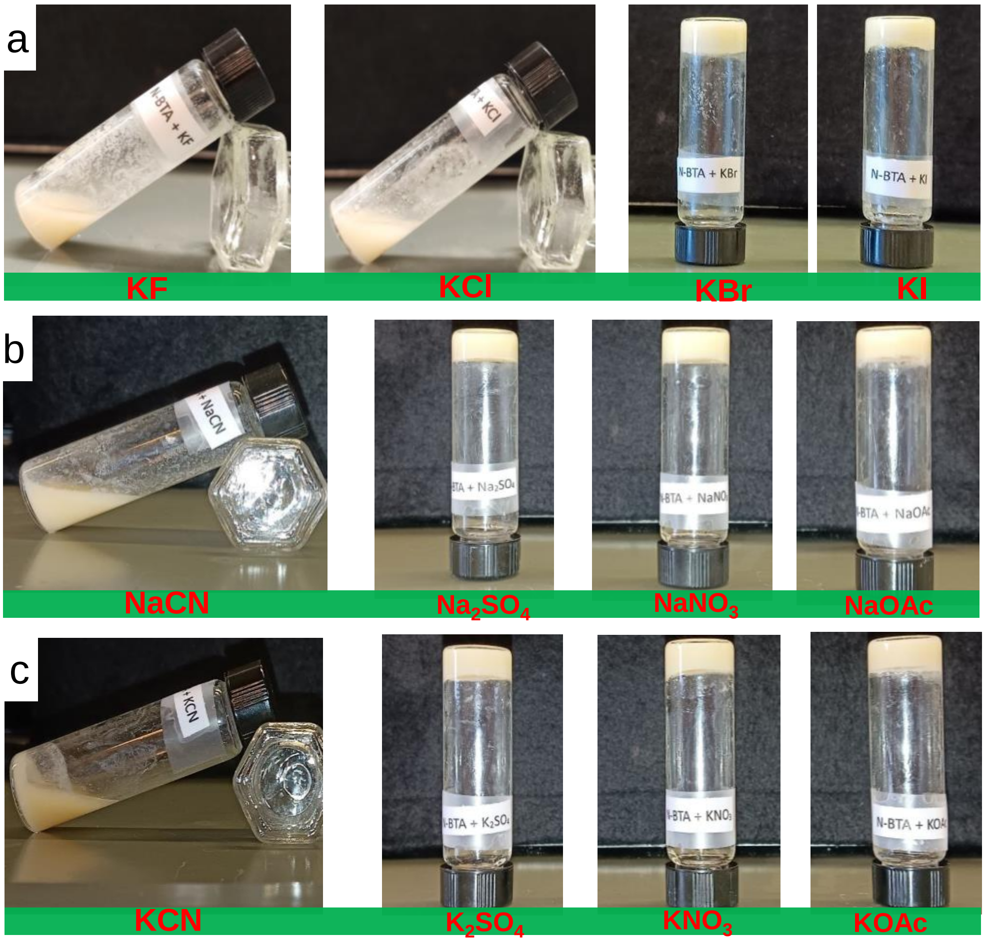

Figure S7. Stimuli-responsive properties of the N-BTA gels with various anions (1.0 eq) at MGC in DMSO/water (1:1, v/v); (a) potassium halides, (b) sodium and (c) potassium salts of cyanide, sulphate, nitrate and acetate anions, respectively.

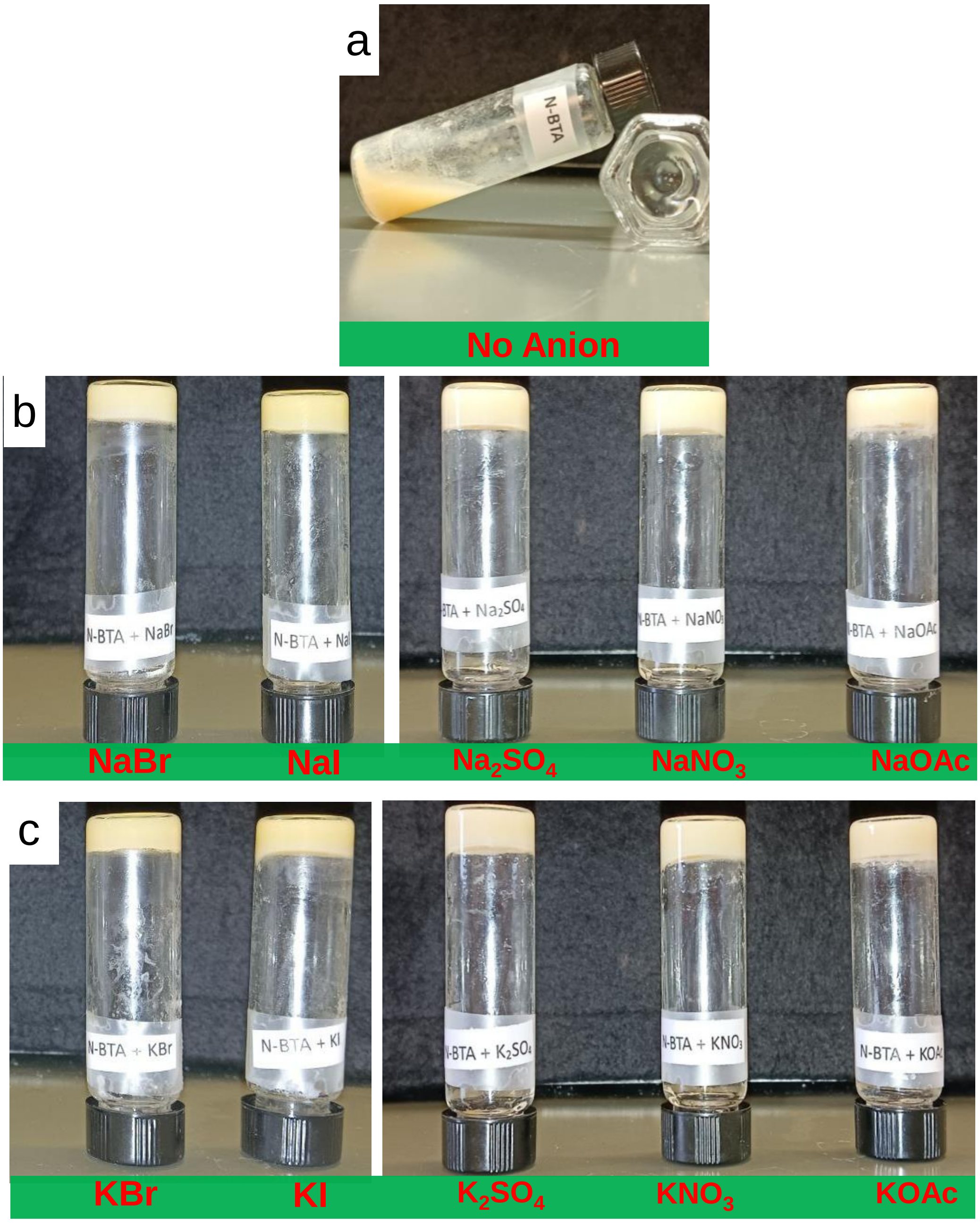

Figure S8. (a) N-BTA gels below MGC in DMSO/water (1:1, v/v) and anion induced gelation with (b) sodium and (c) potassium salts such as bromide, iodide, nitrate, sulphate and acetate ions (1.0 eq.).

Table S2: T_{gel} studies with the gels in DMSO/water (1:1, v/v) in the presence of potassium and sodium salts (1.0 equiv.).

Anion	T_{gel} (°C)	
	Without anion (at MGC)	With anion (at MGC)
KI	98.5	99.6
KBr	98.5	100.3
NaI	98.5	101.6
NaBr	98.5	100.4
K_2SO_4	98.5	98.6
KNO_3	98.5	99.4
CH_3COOK	98.5	103.6
CH_3COONa	98.5	104.5
Na_2SO_4	98.5	100.2
$NaNO_3$	98.5	102.2

6. Dye Adsorption studies

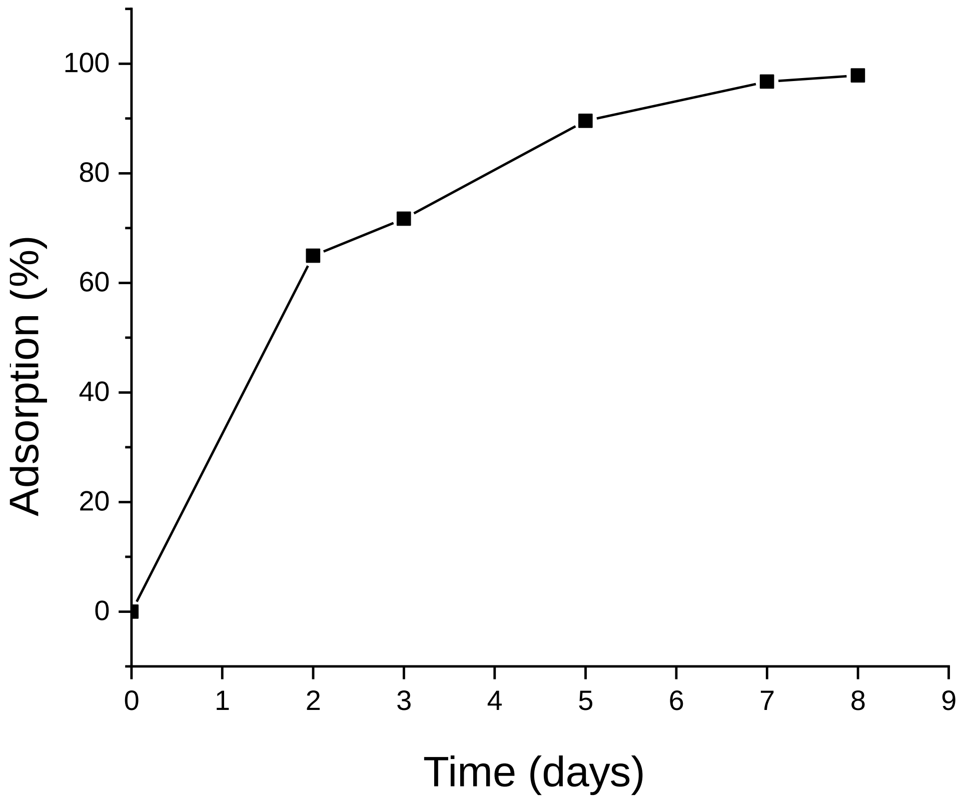

Figure S9. The time-dependent adsorption of MB by N-BTA gel.

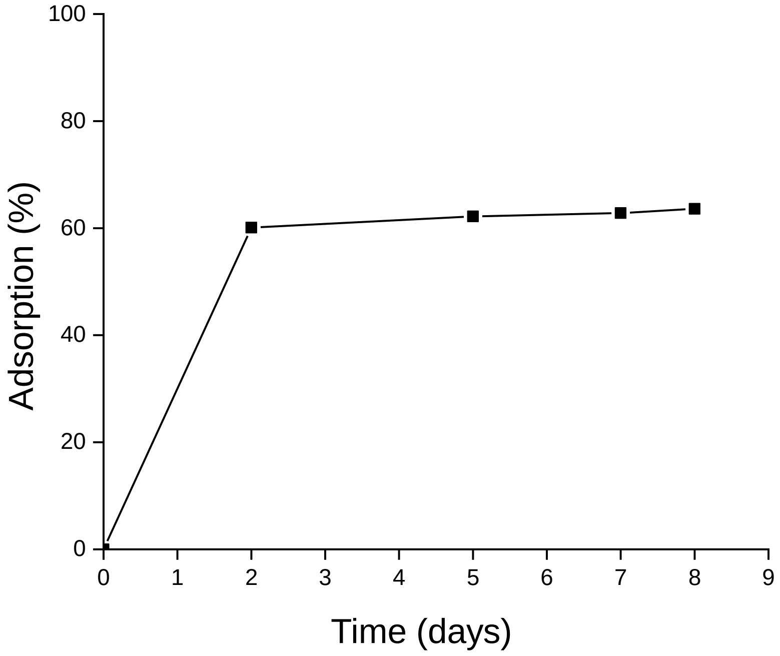

Figure S10. The time-dependent adsorption of MO by N-BTA gel.

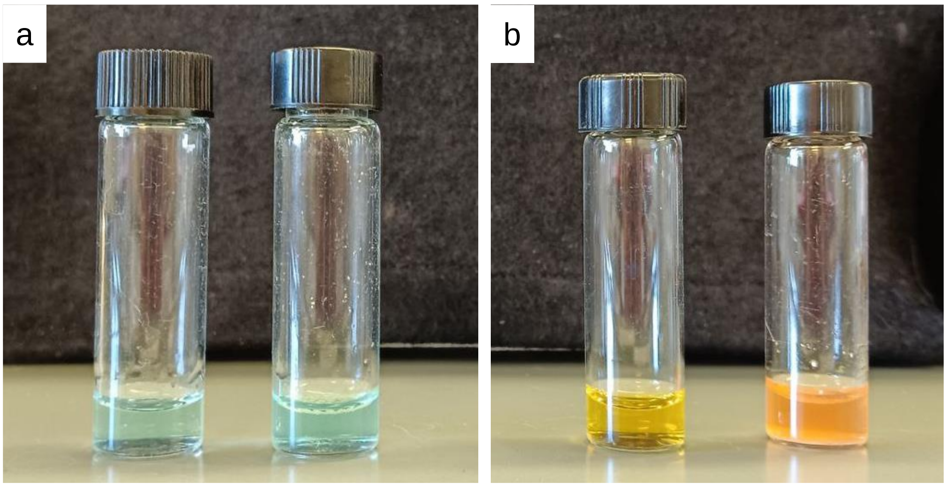

Figure S11. Aqueous solution of (a) MB and MO before and after the addition of dry N-BTA.

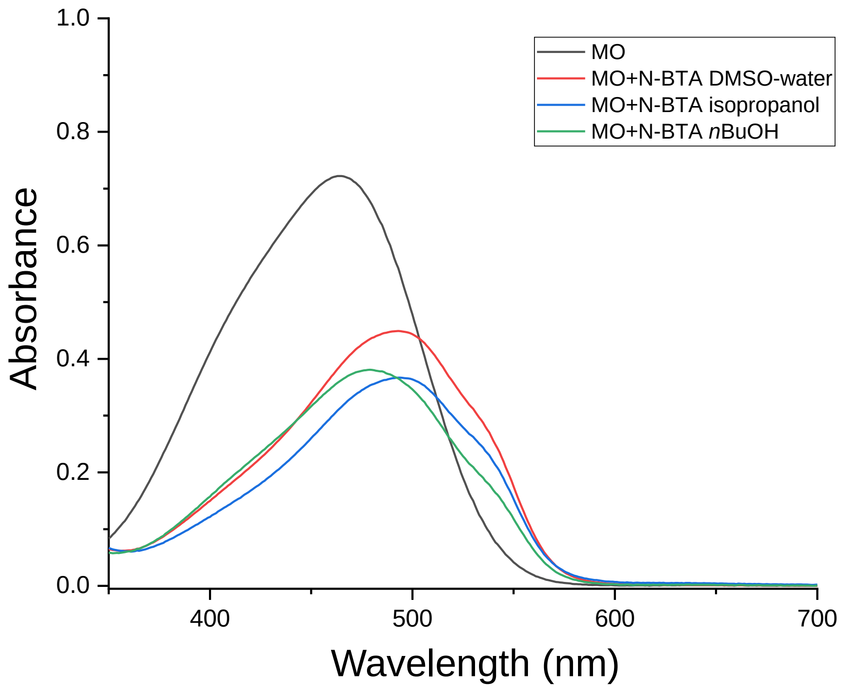

Figure S12. UV-vis experiments of MO (5.0 x 10^{-5} M) with N-BTA gel from n-butanol, isopropanol, and DMSO/water.

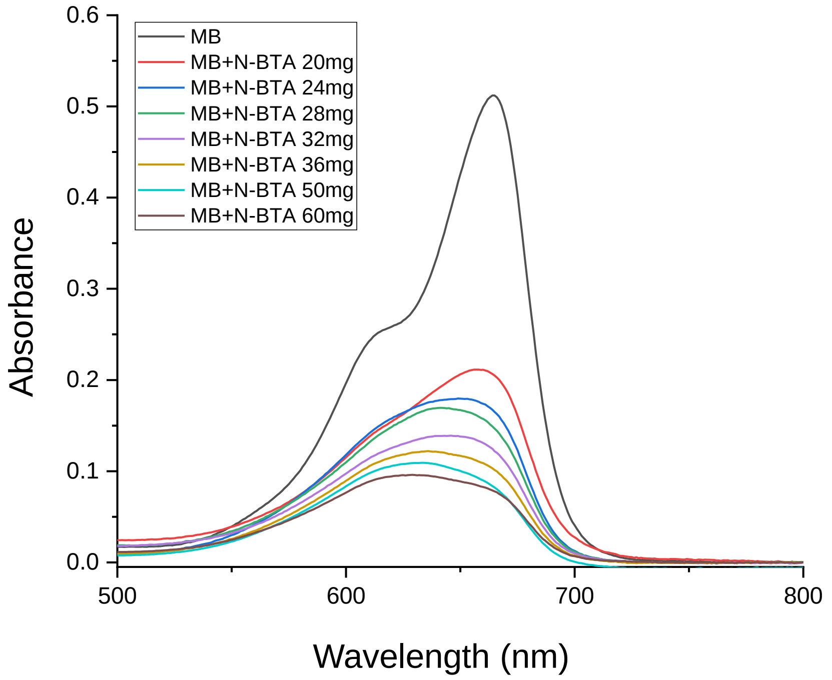

Figure S13. UV-vis experiments of MB (7.5. x 10^{-6} M) with different concentrations of N-BTA after 2 days.

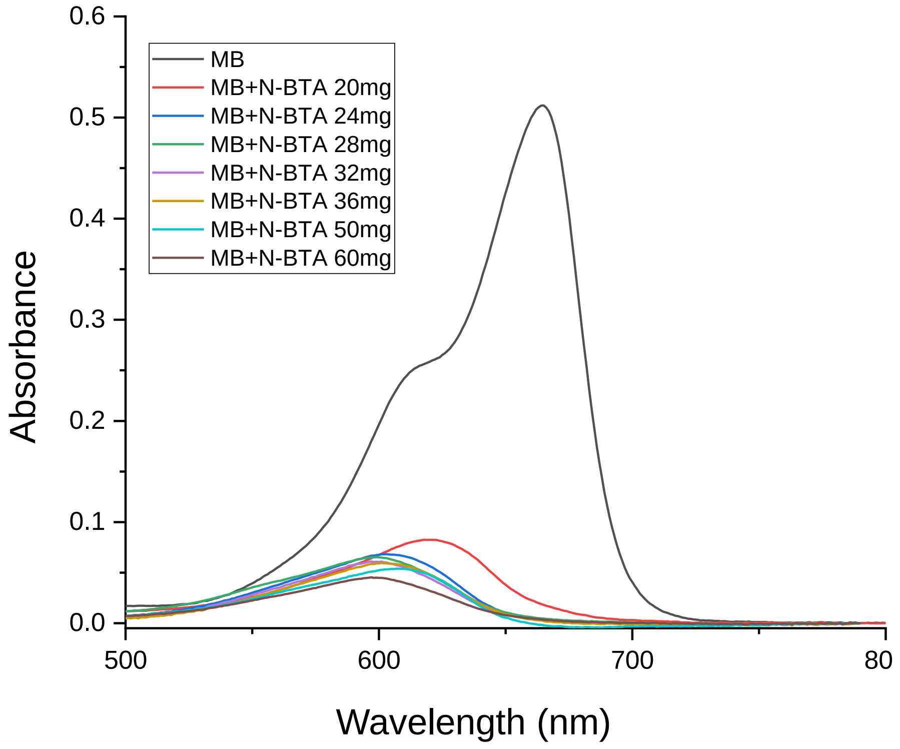

Figure S14. UV-vis experiments of MB (7.5. x 10^{-6} M) with different concentrations of N-BTA after 5 days.

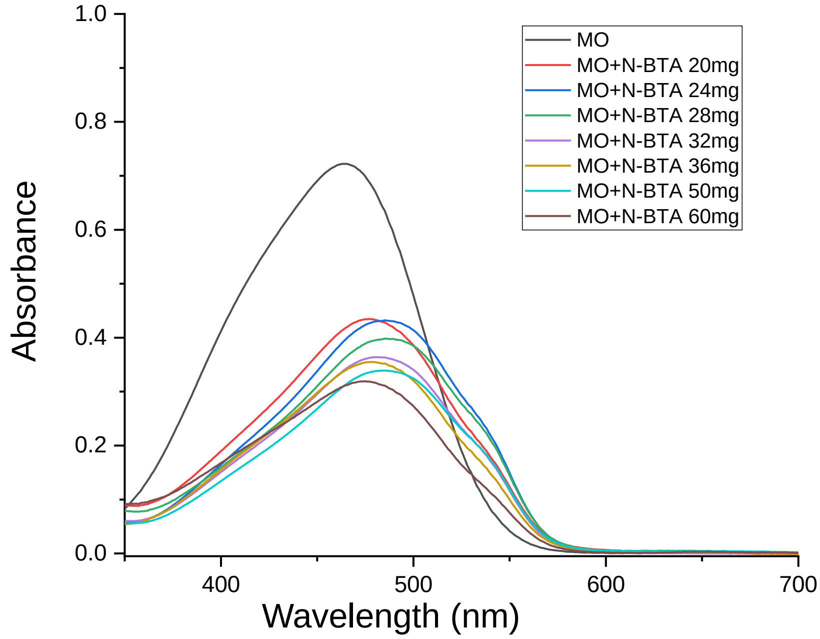

Figure S15. UV-vis experiments of MO (5.0 x 10^{-5} M) with different concentrations of N-BTA after 2 days.

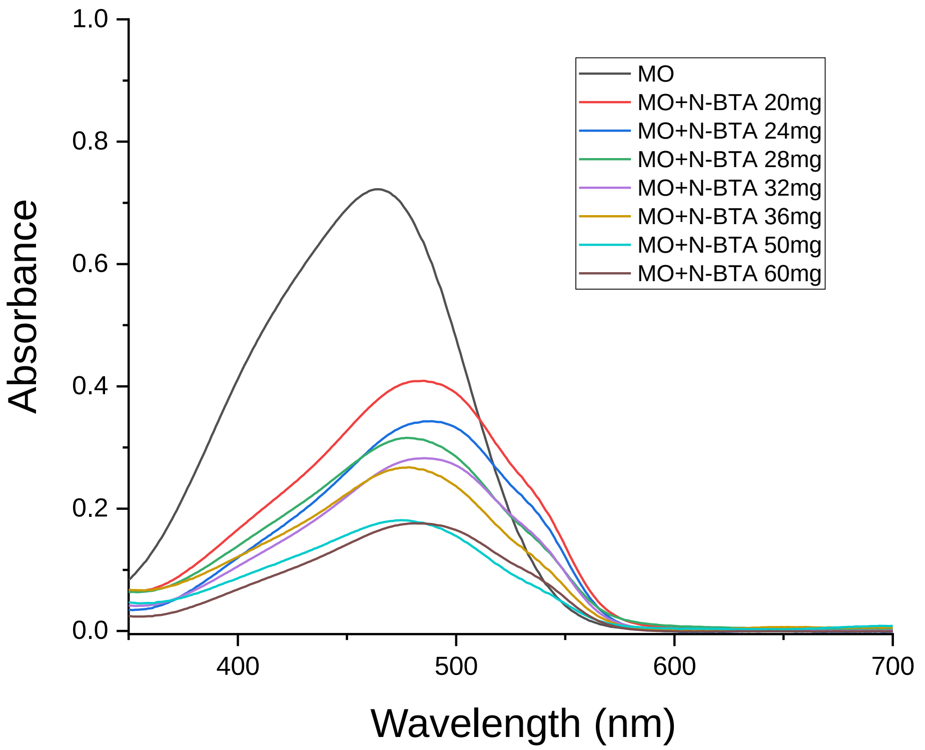

Figure S16. UV-vis experiments of MO (5.0 x 10^{-5} M) with different concentrations of N-BTA after 5 days.

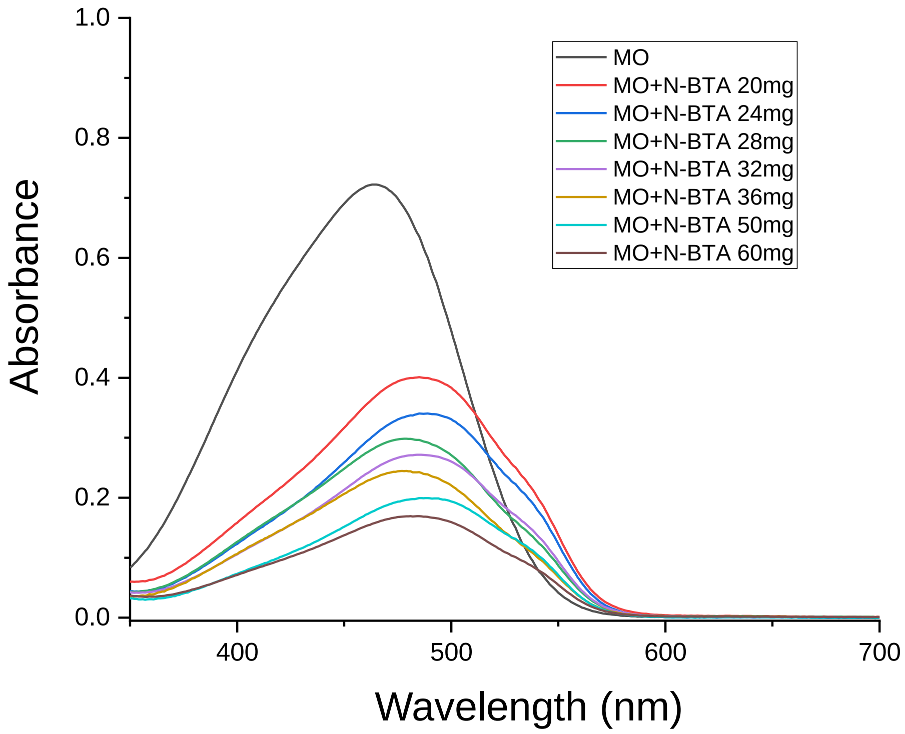

Figure S17. UV-vis experiments of MO (5.0 x 10^{-5} M) with different concentrations of N-BTA after 7 days.

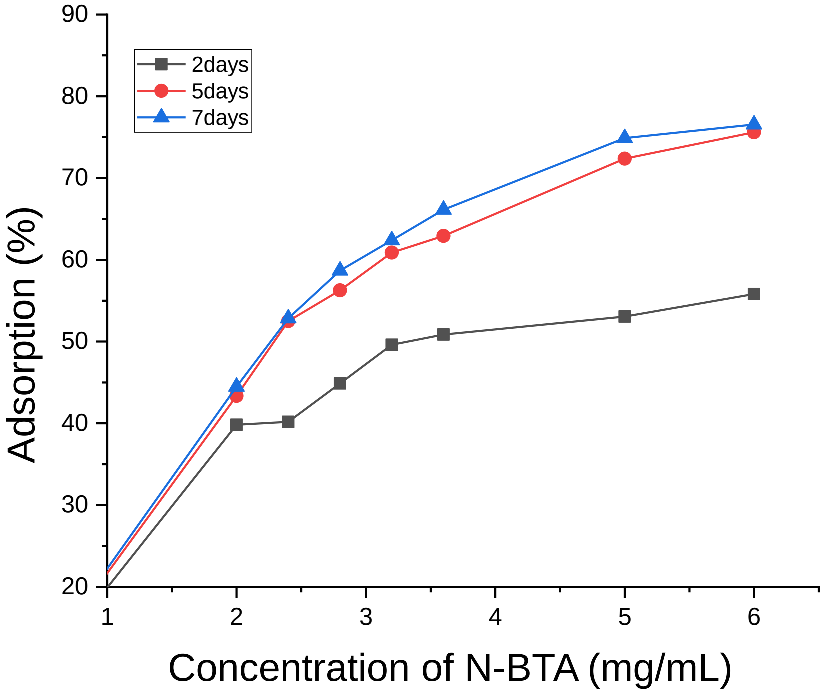

Figure S18. Adsorption ratio of MO with varying concentrations of N-BTA after 2, 5 and 7 days.

7. NMR spectra

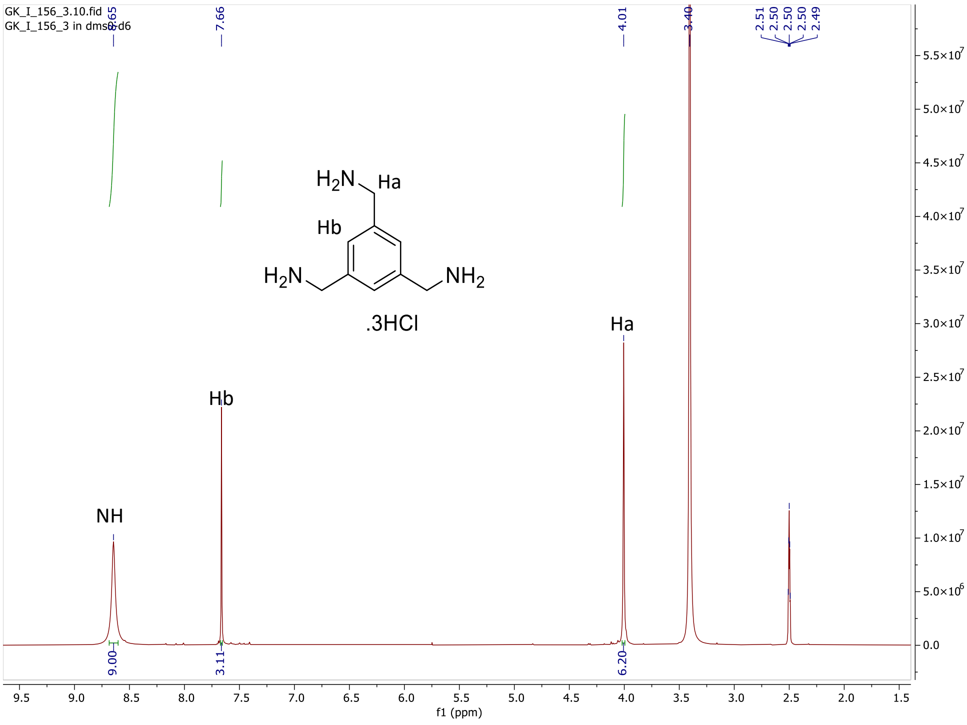

Figure S19. ^{1}H NMR spectrum of benzene-1,3,5-triyltrimethanamine.

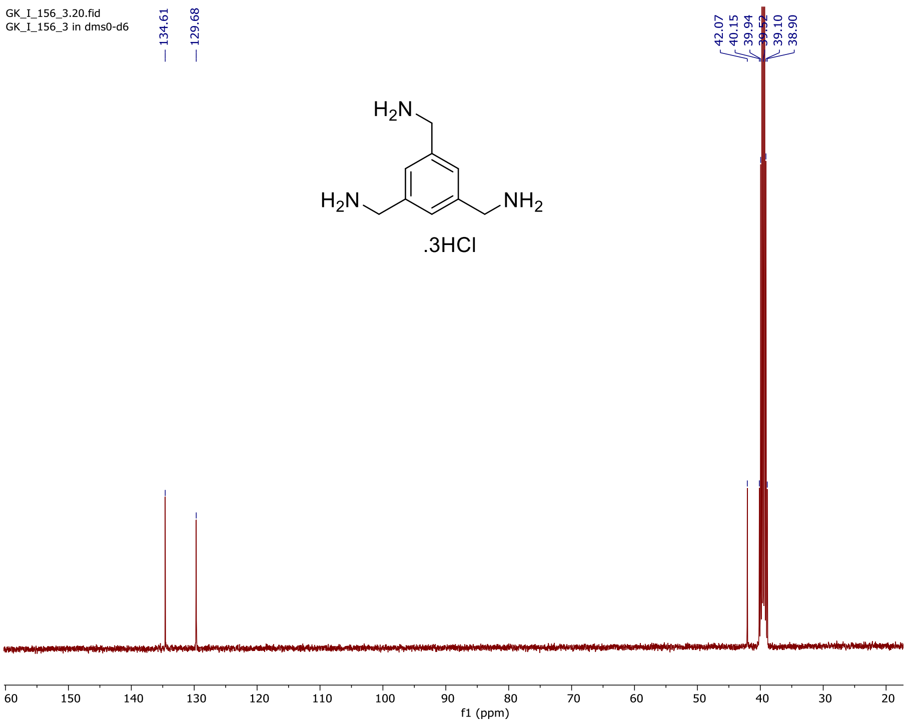

Figure S20. ^{13}C NMR spectrum of benzene-1,3,5-triyltrimethanamine.

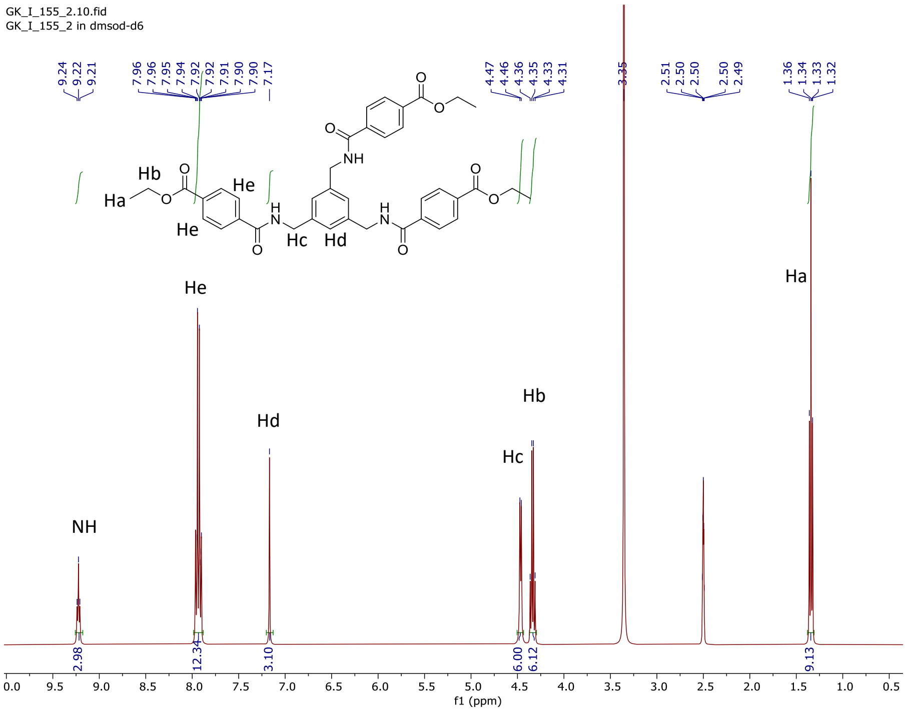

Figure S21. ^{1}H NMR spectrum of N-BTA.

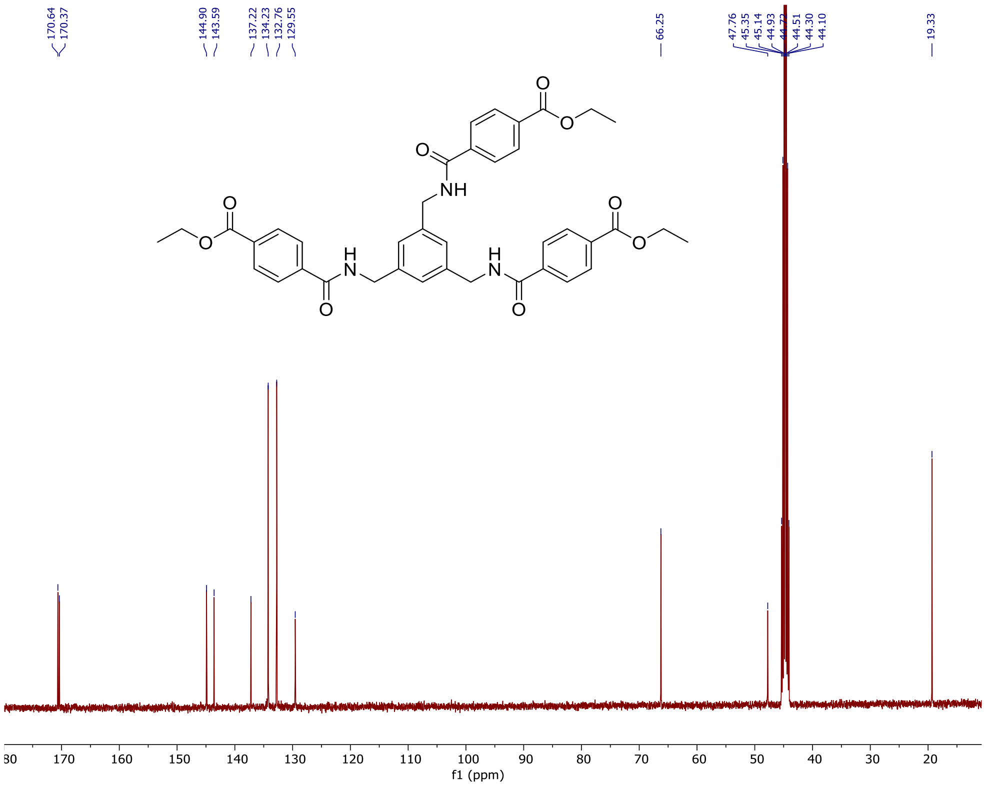

Figure S22. ^{13}C NMR spectrum of N-BTA.
